# Resurrection Flower

**Published:** 1864-04

**Authors:** 


**Resurrection Flower.**—Dr. Deck, of this city, has in his possession an extraordinary floral production. While on a visit to Egypt, inspecting some lead and copper-mines upon the Upper Nile, an Arab was taken ill, and the Doctor rendered him medical aid; and when the Arab recovered, he gave the Doctor this extraordinary plant; and the history furnished of it was, that it was taken from the bosom of an embalmed Egyptian princess, found in one of the vaults containing the remains of Coptic royalty. It is, to all appearance, a dry, dead substance, resembling the flattened head of a poppy, or the cup of an acorn, with a short, woody stem. But upon placing the stem in water, the corolla begins to expand, like a sunflower or dahlia, and in the course of fifteen minutes it will not only unfold, but it will turn its entire leaves backward, until they hang downward in a fringe, like the passion-flower, leaving an exquisite purple heart exposed, and forming a blossom of symmetrical beauty. Since it has been in Dr. Deck's possession it has blossomed some eight or nine hundred times.

Two other specimens of this rare flower are known to exist; one was owned by the celebrated Baron Humboldt, and the other by a distinguished European *savan.* Dr. Deck's rational theory is, that it is a seminal vessel, and may drift about, with its seed carefully folded up, for ages in the desert, and only when it reaches the moisture of an oasis vegetates and blooms.

Not the only specimens, neighbors of the *Banner of Light.* We can illumine you a little, and tell you that the *California Farmer's* collection has *two* specimens of the Resurrection-Flower, and therefore we are as rich as the Baron Humboldt and the distinguished European *savan*, and California will always have her share of rare and beautiful plants from all parts ot the world.—*California Farmer.*

